# Supplementary material for: The Impact of a Change in Employment on Three Work-Related Diseases: A Retrospective Longitudinal Study of 10,530 Belgian Employees
Source: Int J Environ Res Public Health. 2020 Oct 14;17(20):7477. doi: 10.3390/ijerph17207477 (PMC7602491; doi:10.3390/ijerph17207477)
Supplement: Supplementary file 1 [file ijerph-17-07477-s001.pdf]

Supplementary Table S1. List of risk factors
Safety risks and hindrance
Lack of physical exercise
Suffocation in oxygen-poor environment
Conflicts with customers
Stress at work
Burnout
Improper or unacceptable behavior (being victim of violence (physical) and bullying and incivility (non-physical)
Obesity
Noise 87dB
Noise 85dB
Noise 80dB
Physical load
Manual handling of loads
Manual lifting, holding, carrying
Manual pulling and pushing
Manual repetitive tasks
Static load
Shift work without job-specific risks
Shift work with job-specific risks
Night work task-specific particular risks
Night work task-specific particular risks
Mental load due to environmental noise
Psychosocial load
